# Symptom perception and functioning in patients with advanced cancer

**DOI:** 10.1371/journal.pone.0245987

**Published:** 2021-02-04

**Authors:** Eun Joo Yang, Keun Seok Lee, Myong Cheol Lim, Ji Yeon Baek, Ji-Youn Han, Eun-Seung Yu, Seung Hyun Chung

**Affiliations:** 1 Department of Rehabilitation Medicine, Seoul National University Bundang Hospital, Seongnam, Republic of Korea; 2 Department of Internal Medicine, Center for Breast Cancer, Research Institute and Hospital, National Cancer Center, Goyang, Republic of Korea; 3 Department of Obstetrics and Gynecology, Division of Tumor Immunology, Center for Uterine Cancer, and Center for Clinical Trials, National Cancer Center, Goyang, Republic of Korea; 4 Department of Internal Medicine, Research Institute and Hospital, National Cancer Center, Goyang, Republic of Korea; 5 Department of Internal Medicine, Center for Lung Cancer, Research Institute and Hospital, National Cancer Center, Goyang, Republic of Korea; 6 Department of Psychiatry & Behavioral Science, Research Institute and Hospital, National Cancer Center, Goyang, Republic of Korea; 7 Department of Rehabilitation Medicine, Research Institute and Hospital, National Cancer Center, Goyang, Republic of Korea; University of Technology Sydney, AUSTRALIA

## Abstract

**Purpose:**

To explore how symptom perception affects functioning in patients with advanced cancer.

**Materials and methods:**

We conducted a cross-sectional observational study of 459 advanced cancer patients at the national cancer center. Functioning was assessed using the World Health Organization Disability Assessment Schedule (WHODAS) II, and symptoms were evaluated using the Memorial Symptom Assessment Scale-Short Form. Confirmatory factor analysis was conducted to develop a structural model based on different symptom perceptions, such as somatic sensation and experienced symptoms.

**Results:**

The structural model of disability revealed a significant direct pathway involving somatic sensation (β = 16.11, p < 0.001). Experienced symptoms significantly affected somatic sensations (β = 0.717, p < 0.001) but were not directly associated with disability. Unidimensional models exhibited a poor fit. In contrast, a complex model with first-order (somatic sensation) and second-order (experienced symptoms) factors provided an excellent fit, with comparative fit indexes (CFIs) and Tucker Lewis indexes (TLI) of more than 0.950 threshold.

**Conclusions:**

Our findings suggest that relationships to functioning may vary between somatic sensations versus experienced symptoms. The structure of symptoms is best conceptualized by direct somatic sensation and indirect experienced symptoms. A better understanding of symptom perception and the relationship between symptoms and function would facilitate the development of effective rehabilitation programs.

## Introduction

Advanced cancer patients are burdened with both cancer and cancer treatment-related morbidities. Severe disease-related symptoms include nausea, pain, fatigue, lack of energy, and sleep disorders. In addition, chemotherapy-related side effects such as anorexia, nausea, and peripheral neuropathy restrict physical activity. Consequently, patients experience progressively poorer physical function, reduced mobility, and severe restriction of the activities of daily living [1]. The presence of multiple symptoms in patients with advanced cancer [2, 3] may significantly impact functioning [4–7] and quality of life [8–10]. All clinicians involved in the care of patients with cancer should be competent in the assessment and management of symptoms [11].

Physical symptoms are the outcome of perceptual-cognitive processes that detect somatic sensations and interpret them based on experiences [12]. It is unclear whether integrated symptom perception is dependent on functioning. Somatic sensations, such as pain and breathlessness, as well as their interpretation based on experiences (e.g., lack of energy and emotional distress), may have differential impacts on patients’ ability to function [13]. For instance, pain control reduces physical function due to fatigue and drowsiness because fatigue may exacerbate pain and thereby deteriorate functioning. Elucidating the relationship between symptoms and functioning is essential when devising treatment protocols for advanced cancer patients [14].

A model for predicting disabilities in patients with advanced cancer is an unmet clinical need, as it could aid the development of interventions to improve functioning. Here, we used structural equation modeling [15] to evaluate various hypotheses on the latent structures of symptoms related to disability. Three models were examined using data on outpatients with advanced cancer. The first model was a unidimensional model integrating all items that best reflected a single latent construct related to functioning. Symptom numbers may dictate the functioning level. The second model reclassified symptoms into the two dimensions identified in prior studies [8, 16–18]. These dimensions included somatic sensation factors (e.g., aerodigestive, debility, and pain) or symptoms (e.g., fatigue, anorexia, cachexia, and neuro-psychological symptoms). However, a more fine-grained approach may be warranted. Examination of more complex theoretical models is required to understand the underlying perception structure. Structural equation modeling can be used to this end [15]. The third model was complex, and it was unclear whether the clusters were similarly related to functioning. Experienced symptoms may affect functioning only indirectly, unlike somatic sensations. We evaluated the fits of these models to comprehensively explore how symptom perception affected functioning.

## Materials and methods

### Study design

This cross-sectional study was carried out at a single cancer hospital in Korea. Data were collected from patients treated for breast, gynecological, colorectal, and lung cancers. Adult patients (aged 20–64 years) with a diagnosis of advanced cancer, who provided signed informed consent for participation, were included in the study between October 18, 2012 and March 15, 2014. Of 600 advanced cancer patients invited to participate in the study, 470 (78.3%) agreed to be interviewed. After excluding 32 participants with missing data, the data of 438 participants were included in the analyses. The one-to-one structured interviews were conducted by trained interviewers.

### Ethics statement

Participants were provided with a participant information leaflet and written consent was obtained prior to conducting interviews. The present study protocol was reviewed and approved by the Institutional Review Board of National Cancer Center (approval number: NCCNCS-10–375).

### Memorial symptom assessment scale-short form

The Memorial Symptom Assessment Scale-Short Form was used to determine the prevalence and severity of different physical and psychological symptoms experienced by cancer patients [19]. Symptom severity was scored using a four-point scale, with higher scores indicating severe symptoms. The Cronbach’s alpha coefficients ranged from 0.76 to 0.87.

### Korean version of the World Organization Disability Assessment Schedule II

For assessment of disability, we used the interviewer-administered, 36-item questionnaire, which can be used to evaluate health and disability at the population level, or in clinical practice, across a variety of diseases [20]. The 36 questions pertain to the functioning difficulties experienced by the respondent during the previous 30 days. Raw global and domain disability scores were transformed to a 0–100 scale using a previously reported method. Global disability scores were categorized using the International Classification of Functioning (no disability, 0–4; mild disability, 5–24; moderate disability, 25–49; severe disability, 50–100).

### Demographic characteristics

Demographic characteristics included age, sex, marital status (living with or without a spouse), education level (no education or elementary school/middle or high school/university graduate or higher), residential area (rural/urban), and monthly income quartile in US dollars. Employment status was defined as employed (including self-employed) or unemployed (including volunteers, students, homemakers, and retirees). We also obtained information on the site and stage of cancer (primary, recurrent, metastatic, or terminal), treatment (surgery, chemotherapy, radiotherapy, other), and time of diagnosis.

### Data analysis

STATA software was used to perform confirmatory factor analysis comparing the three models that differed in the number of factors and the item-loading patterns. Robust estimation procedures were employed, including the weighted least-squares estimator with SEs, a mean- and variance-adjusted χ2 test that used a full-weight matrix, and the maximum likelihood method with robust SEs. A numerical integration algorithm was employed for maximum likelihood, robust SE model estimation. Numerical integration becomes computationally demanding when estimating models with increasing numbers of factors. Therefore, we used a Monte Carlo method to designate integration points. The number of such points ranged from 5,000 to 10,000. Model fit was assessed using the comparative fit index (CFI), the Tucker Lewis index (TLI), the root mean square error of approximation (RMSEA), and the standardized root mean square residual (SRMR). The χ*2* model reflects the extent to which the data agree with a hypothesis. The CFI and TLI are incremental fit indices that compare an independence model with the hypothesis model [21]. If either index indicated poor model fit, the model was revised using the modification index, which estimates the effect of adding an additional model path to the chi-square statistic [22]. A modification index > 3.84 for a specific path indicates that adding that path significantly improved the fit [22]. The minimal number of cases required per predictor variable was calculated to be 15. Thus, our sample of 459 patients was adequate for SEM.

In all analyses, *p* < 0.05 was taken to indicate statistical significance. The information criteria are relative fit indices of model parsimony that consider model complexity based on the degrees of freedom. Evidence of model fit was accumulated employing standard interpretations of the fit indices, including a χ*2* value that was not statistically significant, CFI and TLI values of at least 0.950, and an RMSEA no greater than 0.080. The SRMR values range from 0 to 1, with values of 0.080 or lower indicating a good fit. The information criteria allow comparisons among non-nested models, with lower values indicating better model fit. Thus, we used the lowest value to determine the optimal fit. Of note, the χ*2* test tends to falsely reject statistical models affording adequate fit if the sample sizes are large. Thus, we preferred the descriptive fit indices when interpreting model fit.

## Results

A total of 459 outpatients were included in the study; 125 (26.6%) were men, and 345 (73.4%) were women. The mean age was 52.3 years (SD, 9.3 years). The age distribution was as follows: < 40 years, n = 45 (9.8%); 40–49 years, n = 121 (26.5%); 50–59 years, n = 181 (39.6%); and ≥ 60 years, n = 110 (24.1%). The most prevalent cancer type was breast cancer (n = 145; 31.7%), followed by gynecological, colorectal, and lung cancers. There were 376 (81.2%) married participants. The majority of participants were living with family or friends (87.6%), and 80 (17.2%) were still in the workforce. Based on a cutoff score of 25, 29.9% of the advanced cancer patients were classified as having a disability. The rate of functioning impairment was highest for participation in society (74.3%) and lowest for self-care (9.4%) (Table 1).

**Table 1:** Demographic characteristics of study participants.

Variables	Category	N	Percentage
Sex	Female	344	73.4
	Male	125	26.6
Age (years)	< 40	46	9.8
	40–49	124	26.4
	50–59	185	39.4
	≥ 60	115	24.5
Type of cancer	Breast	153	32.6
	Gynecological	100	21.3
	Colorectal	117	24.9
	Lung	100	21.3
Marital status	Married	376	81.2
	Not married	87	18.8
Education level	≤ Middle school	134	28.9
	High school	199	42.9
	College	131	28.2
Employment	Yes	80	17.2
	No	385	82.8
Disability	Total score	140^†^	29.9^†^
	Communication	92^†^	19.6^†^
	Getting around	147^†^	31.5^†^
	Self-care	83^†^	17.7^†^
	Getting along	149^†^	32.2^†^
	Life activities	212^†^	45.3^†^
	Participation	346^†^	74.3^†^

^†^Scores ≥ 25 were used to indicate disability based on the WHODAS International Classification of Functioning.

After controlling for the effects of age, sex, employment status, and other symptoms, pain, lack of energy, shortness of breath, and sensitivity were significant predictors of the total disability score, and together explained 46.9% of the variance therein (S1 Table).

The results of confirmatory factor analysis of the evaluated models are shown in Table 2. The one-factor and two-factor models exhibited poor fits as indicated by CFIs and TLIs of less than 0.950 and RMSEAs that exceeded 0.080. By contrast, the complex model exhibited an excellent fit. The CFI and TLIs met the threshold value of 0.950.

**Table 2:** Model fit results as revealed by CFA of symptom and function measures.

Model by sub-study	Log-likelihood	Free parameters, N	CFA results						
			AIC	BIC	χ^2^ value (*df*)	CFI	TLI	RMSEA	SRMR
One-factor	−6,842.7	458	13,820.6	13,932.1	75.17 (27) *	0.955	0.941	0.062	0.039
Two-factor	−6,972.9	458	13,993.2	14,108.8	245.74 (26) *	0.797	0.719	0.136	0.185
Complex	−6,875.6	459	13,807.3	13,922.9	59.83 (26) *	0.969	0.957	0.053	0.034

AIC, Akaike information criterion; BIC, Bayesian information criterion; CFA, confirmatory factor analysis; CFI, confirmatory fit index; RMSEA, root mean square error of approximation; TLI, Tucker/Lewis index; SRMR, standardized root mean square residual.

^†^The models tested included a unidimensional one-factor model, the experienced symptom (ES) and somatic sensation (SS) two-factor model, and a complex model with first-order SS factors and two second-order ES factors.

The exogenous variable in all three models was the disability score.

Endogenous variables were the total symptom score in the one-factor model and two latent domains (e.g., ES and SS) in the two-factor and complex models.

* *p* < 0.001.

**Fig 1:**
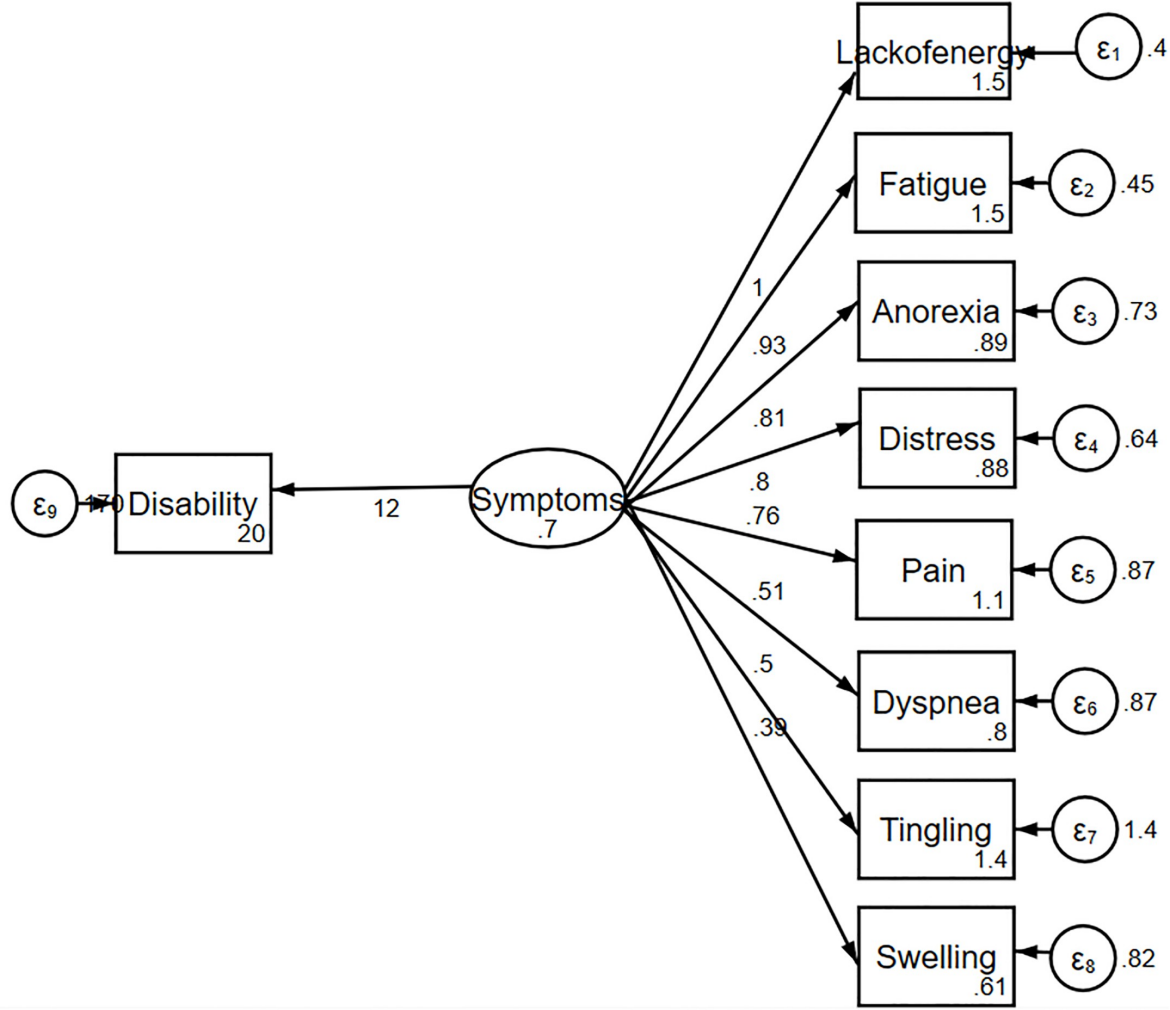
One-factor model of the relationship between symptoms and disability.

**Fig 2:**
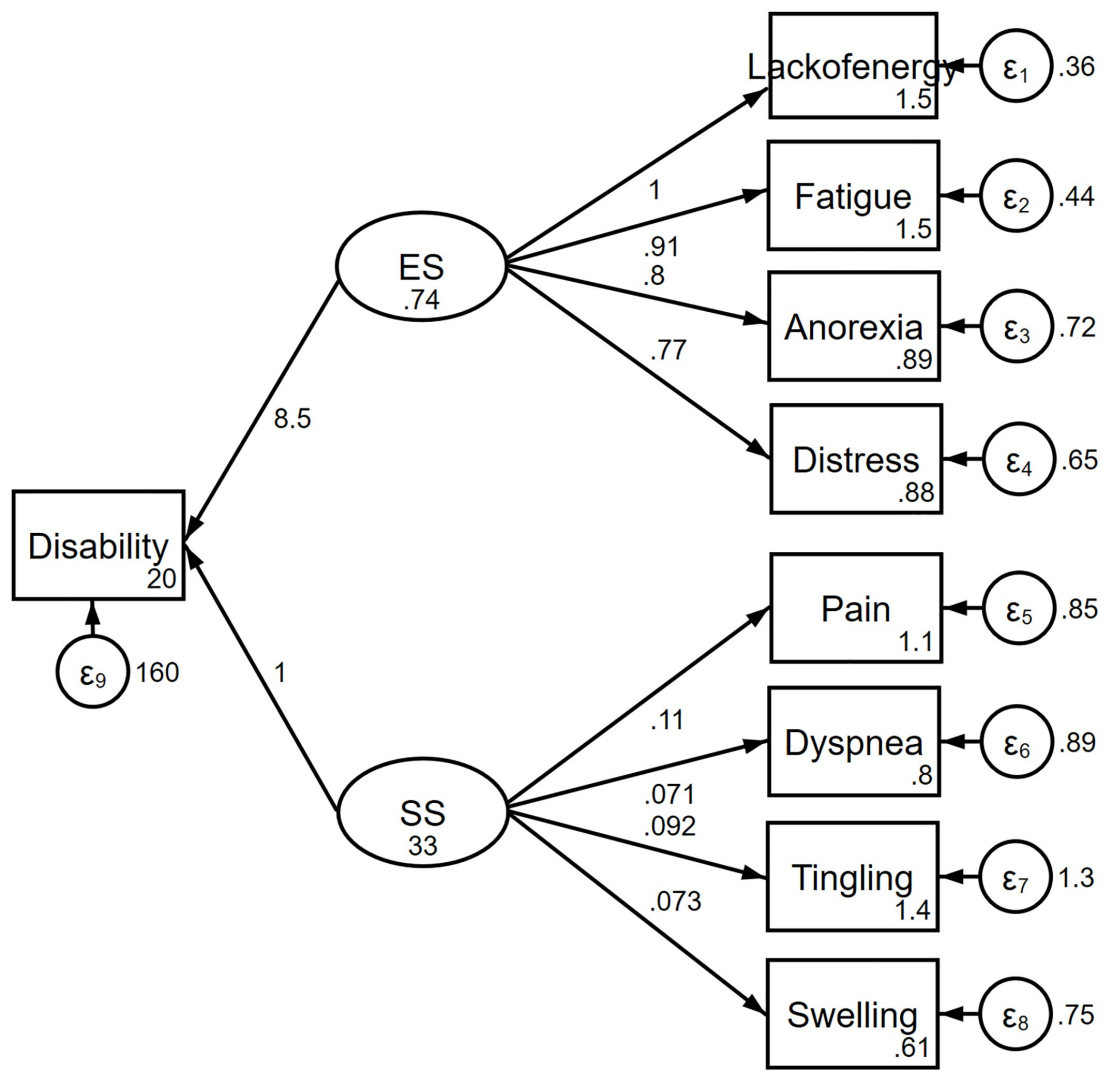
Two-factor model of the relationship between symptoms and disability. ES, experienced symptoms; SS, somatic sensation.

**Fig 3:**
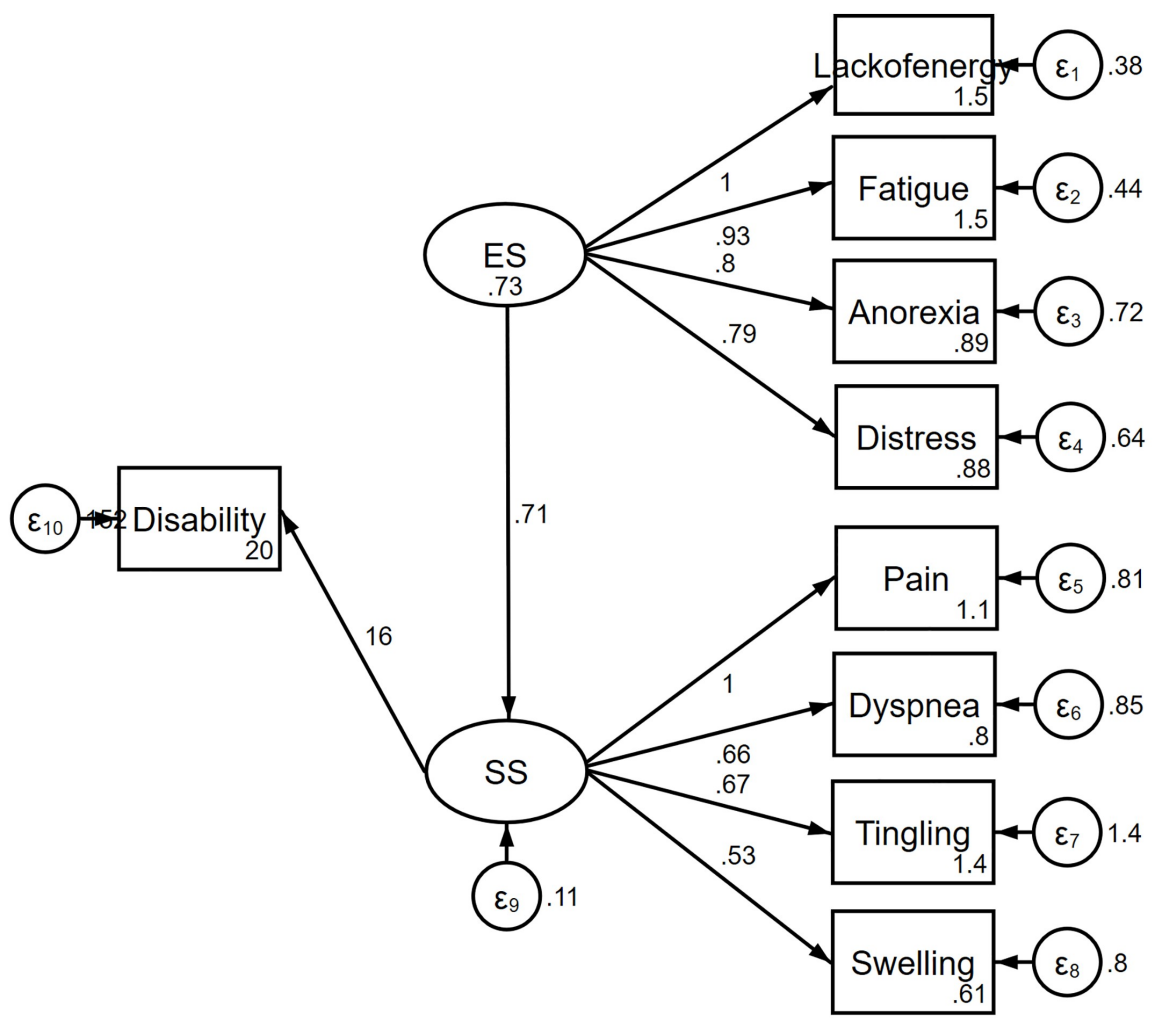
Complex model of the relationship between symptoms and disability. ES, experienced symptoms; SS, somatic sensation.

The information criteria favored the complex model over the one-factor (Fig 1) and two-factor models (Fig 2). The complex relationships between symptoms and disability domain scores are illustrated (Fig 3). The values shown next to the single-headed arrows are the estimated standardized regression coefficients. The experienced symptoms significantly affected somatic sensation (β = 0.717, 95% CI 0.591–0.842, *p* < 0.001). Lack of energy exhibited the largest direct association with the experienced symptoms; pain had the largest direct association with somatic sensation. All coefficients were statistically significant (*p* < 0.05). Somatic sensation was significantly associated with the total disability score (β = 16.11, 95% CI 13.84–20.38, *p* < 0.001); however, experienced symptoms were not associated with the total disability score.

## Discussion

We explored how symptom perception affected functioning in patients with advanced cancer. The symptom clusters were not similarly related to functioning. Experienced symptoms may independently reinforce the effects of sensational perception on the functioning of patients with advanced cancer, whereas somatic sensation directly influences functioning. The differences highlighted the importance of a multidimensional approach.

**Fig 4:**
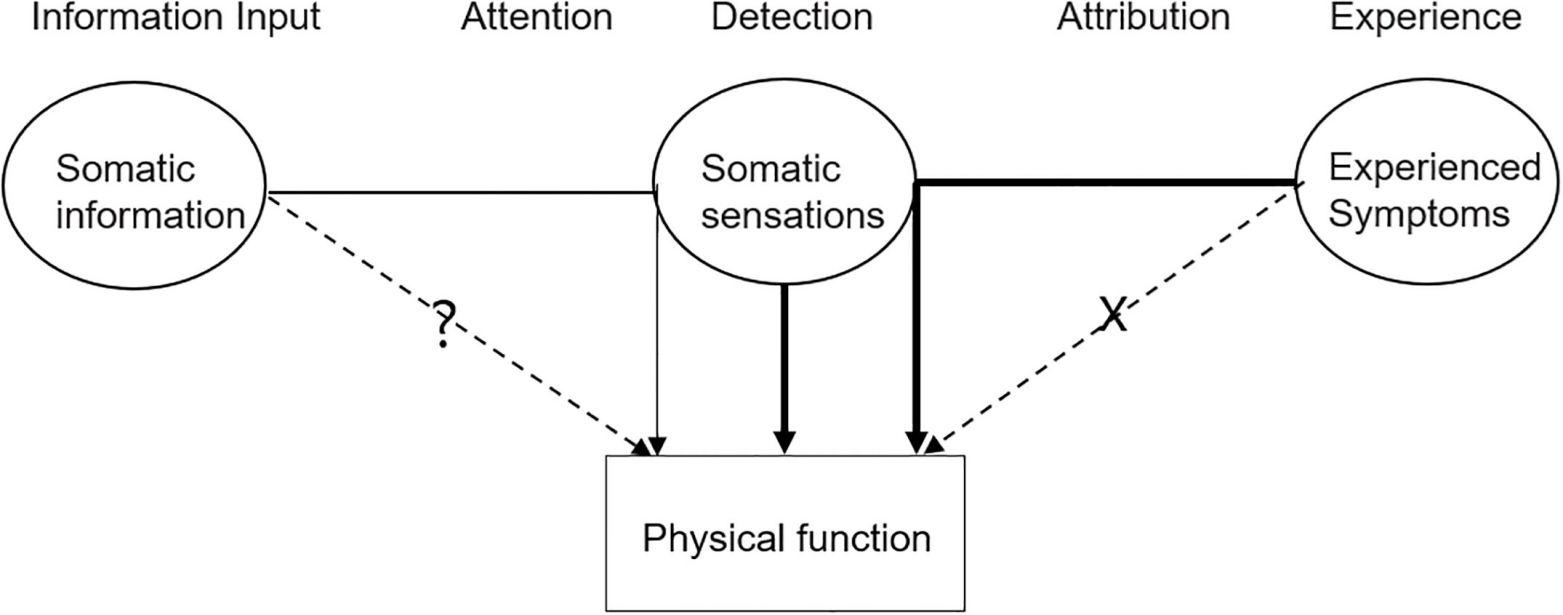
Simplified symptom perception-function model.

Symptoms can be divided into somatic sensation and experienced symptoms according to the mechanisms underlying individual symptom domains. The effects of symptoms on function are two-fold. Somatic sensation (e.g., pain) has deteriorated functioning directly. Experienced symptoms (e.g., fatigue and distress) are more complex than somatic sensation, and their interpretation relies on daily life experiences. We found that experienced symptoms have affected functioning by modifying the intensity of somatic sensations, as summarized in the model of Fig 4.

Effective management requires a better understanding of the ways in which symptoms are perceived and how this influences functioning. The current study found a distinction between somatic sensations versus experienced symptoms with regard to their relationships to functioning. Previous studies have sought correlations between symptoms and functioning by deriving total scores or those of broad symptom cluster dimensions, precluding detection of domain-specific correlations. However, the distinction of these domains revealed distinct correlations among domains, highlighting the need for further investigation. Miaskowski and Lee [23] reported that the symptom cluster of insomnia, pain, and fatigue had a consistent effect on patients’ functioning. We found that pain exhibited the strongest direct association with somatic sensation, directly affecting the disability level. Vainio *et al*. [24] reported that only pain had a significant relationship with performance status. However, this effect was associated with experienced symptoms of fatigue or anorexia. Pain control trials commonly use total pain scores as outcome measures. This affords distinct statistical advantages (e.g., use of a small sample, high power, and reduced type I error); however, such an approach does not adequately capture the complexity of the construct. When calculating domain scores, we recommend the use of mean values rather than sums. Symptoms should be controlled after considering the complex effects of control on other symptoms indirectly related to the functioning.

This study had several limitations. First, the results may have limited generalizability because the study subjects were recruited from a single national cancer center. Furthermore, advanced cancer patients aged over 80 years were not included. To improve the generalizability of the findings, socially isolated and older advanced cancer patients should be included in future studies. Second, we did not conduct subgroup analyses according to the type of primary cancer. Understanding the barriers to functioning in patients with specific types of cancer may be useful for the development of rehabilitation programs aimed at improving functioning. Finally, this was a cross-sectional study, so causal relationships could not be established.

## Supporting information

S1 Table Correlations between symptoms and functioning domains after controlling for other symptoms in multiple regression analyses. (DOCX)

S1 Data (XLSX)
