# Supplementary material for: Symptom perception and functioning in patients with advanced cancer
Source: PLoS One. 2021 Feb 4;16(2):e0245987. doi: 10.1371/journal.pone.0245987 (PMC7861403; doi:10.1371/journal.pone.0245987)
Supplement: S1 Table — (DOCX) [file pone.0245987.s001.docx]

**S1 Table. Correlations between symptoms and functional domains after controlling for other symptoms in multiple regression analyses.**

| Symptoms |  | Correlation coefficient | | | | | | |
| --- | --- | --- | --- | --- | --- | --- | --- | --- |
|  | *n*^†^ (%) | Total disability | communicating | Getting around | Self-care | Getting along | Life activities | Participation |
| 1. Difficulty concentrating | 16 (3.4) | 1.045 | 4.403** | 0.657 | 0.475 | 2.274 | –0.338 | –1.181 |
| 2. Pain | 65 (13.9) | 2.300** | 1.465* | 4.469** | 1.432* | 0.826 | 3.692* | 1.777** |
| 3. Lack of energy | 74 (15.8) | 2.141* | 0.653 | 3.650* | 2.063* | 1.3910 | 3.113 | 2.165 |
| 4. Cough | 27 (5.8) | –0.949 | 0.145 | –0.022 | –0.683 | –1.170 | –2.334 | –1.411 |
| 5. Feeling irritable | 66 (14.1) | –0.456 | –0.951 | –1.670 | –0.429 | –1.478 | –0.265 | 1.948 |
| 6. Dry mouth | 35 (7.5) | 1.732 | 1.924 | 2.503 | 1.330 | 1.306 | 2.411 | 1.508 |
| 7. Nausea | 32 (6.9) | –0.910 | –1.130 | 0.481 | –0.308 | –0.127 | –2.260 | –2.210 |
| 8. Feeling drowsy | 49 (10.5) | –0.629 | –1.552 | 0.325 | 0.025 | –1.597 | –1.203 | 0.200 |
| 9. Tingling | 100 (21.4) | –0.028 | –0.713 | 1.744* | 0.265 | –1.507 | –0.165 | 0.275 |
| 10. Difficulty sleeping | 68 (14.6) | 0.212 | 0.412 | –0.406 | –0.371 | 0.920 | 1.191 | –0.333 |
| 11. Feeling bloated | 42 (9.0) | 0.667 | 1.973 | –0.551 | –0.807 | 0.942 | 2.764 | –0.3193 |
| 12. Dysuria | 41 (8.8) | 1.659 | 0.629 | 3.006 | 2.007* | 0.876 | 1.329 | 2.086 |
| 13. Vomiting | 15 (3.2) | 0.684 | –0.082 | 1.234 | 1.291 | –1.360 | 0.756 | 2.140 |
| 14. Dyspnea | 41 (8.8) | 1.592* | 1.215 | 1.833 | 1.100 | 1.842 | 1.189 | 2.3789* |
| 15. Diarrhea | 13 (2.8) | –2.766 | –0.998 | –3.281 | –3.701 | –1.989 | –3.657 | –2.980 |
| 16. Feeling sad | 43 (9.2) | 0.968 | –0.162 | –0.946 | –0.837 | 3.931* | 2.056 | 1.311 |
| 17. Sweats | 31 (6.7) | 0.018 | –0.973 | –0.561 | 0.472 | 1.639 | 0.769 | –0.919 |
| 18. Worrying | 71 (15.3) | –0.626 | –0.373 | –2.523 | –0.893 | –0.682 | 0.620 | 0.492 |
| 19. Sexual problem | 19 (4.1) | –1.616 | –2.427 | –3.303 | –0.808 | 0.677 | –2.784 | –1.308 |
| 20. Itching | 20 (4.3) | 0.285 | 1.612 | 0.526 | 0.479 | 0.680 | –0.924 | –0.382 |
| 21. Anorexia | 45 (9.6) | 0.153 | 1.198 | –1.359 | 0.145 | –0.117 | –0.940 | 1.863 |
| 22. Dizziness | 26 (5.6) | –0.335 | –1.235 | 0.976 | 0.379 | 0.707 | –0.822 | –2.098 |
| 23. Difficulty swallowing | 11 (2.6) | 1.398 | –0.487 | 0.568 | 3.779* | 2.331 | 2.039 | 0.326 |
| 24. Sensitivity | 48 (10.3) | 2.335* | 2.963* | 3.237 | 1.489 | 4.422* | 0.642 | 1.223 |
| 25. Fatigue | 84 (17.9) | –0.259 | –0.968 | 0.914 | –0.137 | –0.907 | –0.422 | –0.026 |
| 26. Distress | 43 (9.2) | 0.908 | 0.783 | 0.726 | 0.826 | –0.915 | 0.528 | 3.459* |
| 27. Forgetfulness | 45 (9.6) | 0.773 | 2.482* | 0.048 | –0.534 | –1.135 | 3.700* | –0.015 |
| 28. Mouth ulcer | 31 (6.6) | –1.279 | 0.601 | –2.102 | –0.046 | –2.921 | –3.167* | –0.592 |
| 29. Tastes | 46 (9.9) | 1.513 | 0.658 | 1.811 | 1.554 | 2.357 | 3.124* | –0.511 |
| 30. Weight loss | 25 (5.4) | –0.576 | –0.589 | 0.103 | 0.738 | –1.436 | –0.426 | –1.673 |
| 31. Constipation | 34 (7.3) | –1.537 | –0.786 | –0.964 | –0.382 | –2.876 | –1.737 | –2.691 |
| 32. Swelling | 31 (6.6) | 1.116 | –0.939 | 1.948 | 1.075 | 1.234 | 2.458* | 1.772 |
| 33. I don’t look such as myself | 15 (3.2) | 4.306 | 3.701 | 2.886 | 3.939 | 5.929 | 6.511 | 2.649 |
| 34. Skin changes | 33 (7.1) | 0.111 | 0.895 | 0.030 | –0.950 | 1.096 | –0.575 | –0.062 |
| *r*^2^ |  | 0.469 | 0.409 | 0.367 | 0.374 | 0.318 | 0.374 | 0.347 |

**p* < 0.05; ***p* < 0.001

^†^The optimal symptom score cutoff was 3.
